# Implications of COVID-19 in Acute Mesenteric Ischemia and Bowel Necrosis: A Case Report

**DOI:** 10.7759/cureus.47867

**Published:** 2023-10-28

**Authors:** Kelsey R Burrows, David L Remington, James J Cappola

**Affiliations:** 1 General Surgery, Campbell University School of Osteopathic Medicine, Lillington, USA; 2 General Surgery, University of North Carolina (UNC) Wayne, Goldsboro, USA; 3 Internal Medicine, Campbell University School of Osteopathic Medicine, Lillington, USA

**Keywords:** acute mesenteric ischemia, covid-19, bowel necrosis, short gut syndrome, thromboembolic disease, hypercoagulability

## Abstract

Severe acute respiratory syndrome coronavirus 2 (SARS-CoV-2) is known to provoke a state of hypercoagulability that may lead to devastating consequences. This has been well established since the onset of the coronavirus pandemic in 2019; however, the specific relationship between COVID-19 and thrombus formation remains poorly understood. There has been increasing documentation of gastrointestinal (GI) complications in patients infected with the virus, including potentially lethal acute mesenteric ischemia (AMI), regardless of prior history of GI disease or risk factors for hypercoagulable states. Not only is mesenteric ischemia difficult to diagnose but it is also associated with high rates of morbidity and mortality, warranting prompt identification and treatment to improve clinical outcomes.

We herein present a case of diffuse intestinal necrosis secondary to mesenteric thrombus formation in a previously healthy female five days after the resolution of her COVID-19 symptoms. The high rates of morbidity and mortality linked to AMI underpin the need for clinicians to maintain a high index of suspicion for thrombotic complications of COVID, even in healthy patients. This case emphasizes the importance of a thorough history-taking, physical examination, and laboratory workup even in patients without a current COVID-19 infection or predisposing thrombotic risk factors. Additionally, it suggests that the hypercoagulable state associated with a COVID-19 infection may persist after the primary COVID-19 symptoms have resolved.

## Introduction

In December 2019, the first case of SARS-CoV-2 was reported in Wuhan, China. Since then, there have been over 770 million confirmed cases and nearly 7 million deaths as of September 6, 2023, according to the World Health Organization [1].

Severe acute respiratory syndrome coronavirus 2 (SARS-CoV-2) infects human cells by binding to the angiotensin I-converting enzyme 2 (ACE2) receptor, found throughout the body in the lungs, liver, kidneys, and intestines. Binding to the ACE2 receptor causes direct damage to the vessel wall and promotes thrombus formation, a process explained by Virchow’s triad [2]. While most patients with COVID-19 experience respiratory symptoms, there are increasing reports of patients experiencing gastrointestinal (GI)-dominant manifestations similar to those of viral gastroenteritis, such as abdominal pain, nausea, vomiting, and diarrhea. A cross-sectional, multicenter study conducted in China found that 50.5% of the total 204 patients with COVID-19 included in the study reported GI complaints [3]. In six of those cases, patients reported GI symptoms in the absence of respiratory symptoms [3]. The presence of GI manifestations in patients with a current or past COVID-19 diagnosis should prompt clinicians to think of the thromboembolic effects of COVID-19, as these sequelae may be deadly if not identified in a timely manner.

## Case presentation

**Figure 1:**
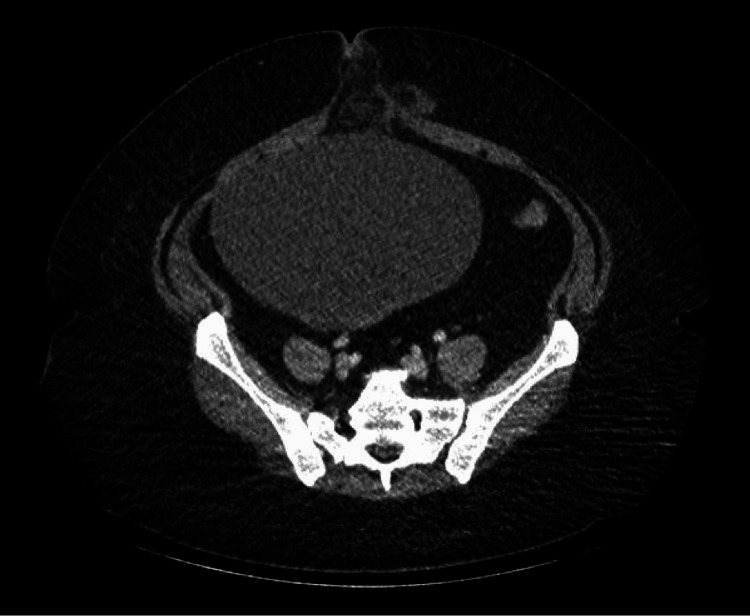
Abdominal CT scan showing a large cystic mass with an overlying fat-containing umbilical hernia

**Figure 2:**
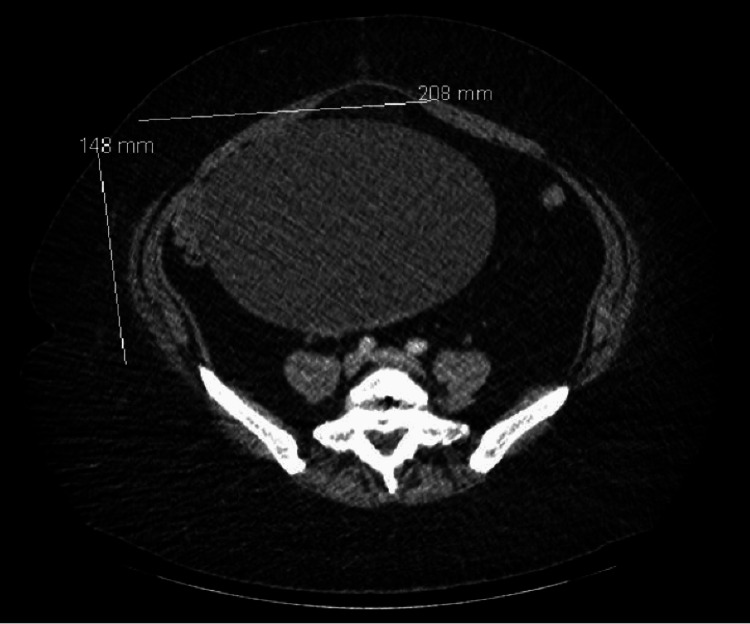
Abdominal CT scan showing a large cystic mass measuring 20.8 x 14.8 cm located in the right adnexal region

A previously healthy 49-year-old woman presented to the emergency department with abdominal pain and associated nausea, vomiting, and diarrhea for six hours. Two weeks prior to presentation, she had tested positive for COVID-19 and recovered at home with supportive care. A physical exam revealed a soft, nondistended abdomen with tenderness in the lower abdomen and periumbilical region. No masses were palpable. Laboratory workup revealed leukocytosis (white blood cells (WBC): 14.5x10^9^/L, absolute neutrophil count: 12.9x10^12^/L), while comprehensive metabolic panel, lipase, and magnesium levels were unremarkable. Inflammatory markers, lactic acid level, arterial blood gas (ABG), and coagulation panel studies were not performed during the initial laboratory evaluation. Abdominal computed tomography (CT) scans (Figures 1-2) revealed a large cystic mass measuring 20.8 x 14.8 cm located in the right adnexal region, with an overlying fat-containing umbilical hernia.

**Figure 3:**
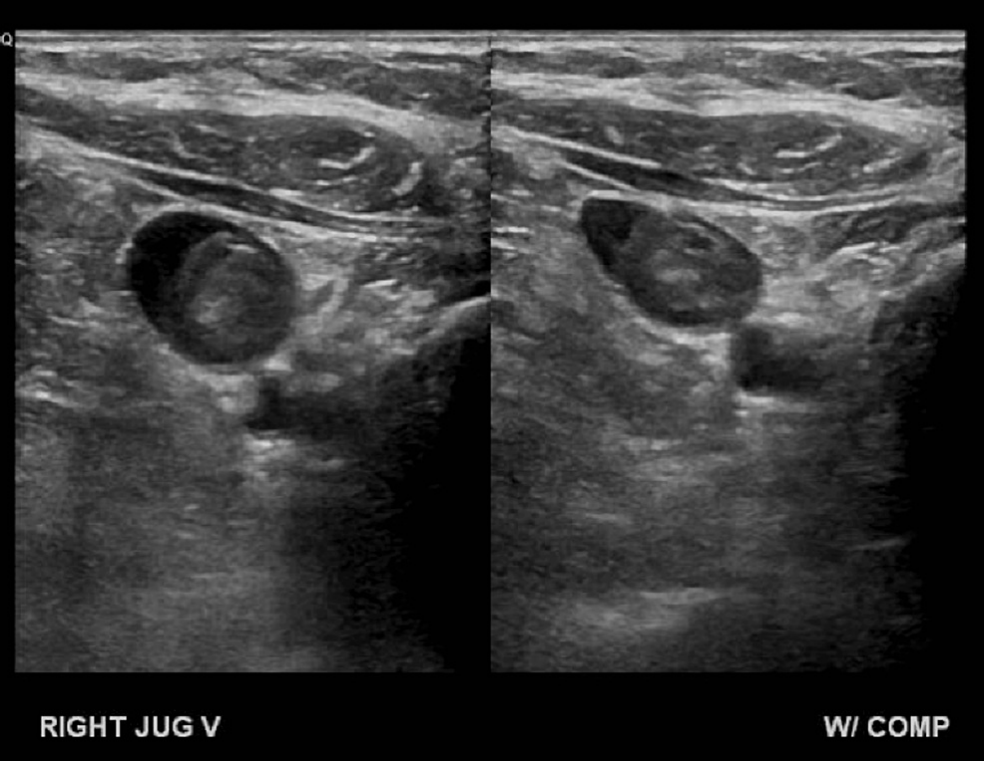
Duplex ultrasound of the right upper extremity shows right jugular vein thrombosis.

Fluid was present within the hernia, likely due to the presence of some mesenteric vessels within the hernia sac. The small bowel and root of the mesentery were benign in appearance. The patient was initially discharged with oxycodone and ondansetron with instructions to follow up for outpatient elective surgery to drain the ovarian cyst. However, she returned four hours after discharge with unrelenting abdominal pain. A complete blood count and basic metabolic panel were performed at this time and demonstrated worsening leukocytosis (WBC: 22.8×10^9^/L). Prior to surgery, no additional labs were obtained. She was taken to the operating room for a diagnostic laparoscopy converted to an exploratory laparotomy due to visual obstruction by the ovarian cyst. Drainage of the cyst revealed clear, straw-colored fluid. The small bowel could then be identified and was found to be profoundly ischemic with areas of necrosis. A thorough bowel inspection revealed the entire small intestine and cecum to be profoundly ischemic and necrotic. A point of viable jejunum was identified approximately 15 cm distal to the ligament of Treitz. The majority of the small bowel was resected, along with a right hemicolectomy and a right salpingo-oophorectomy. The bowel was left in discontinuity. A surgical pathology report identified focal mesenteric intravascular thrombosis and a benign ovarian serous cystadenofibroma. Heparin was given on postoperative day one and continued until discharge. The patient recovered well with physical therapy, total parenteral nutrition, and pain medication. Fifteen days after surgery, this patient was discharged with a portacath, a percutaneous endoscopic gastrostomy (PEG) tube, a referral for a small bowel transplant, and instructions to follow a full liquid diet in addition to total parenteral (TPN) infusions. About two months after discharge, the patient returned with sepsis and hypovolemic shock. During this hospital stay, bowel continuity was restored with a jejunocolostomy. Additionally, she developed right upper extremity pain and swelling. Doppler ultrasound (Figure 3) revealed right internal jugular and subclavian vein thrombosis.

She had no personal or family history of deep vein thrombosis or pulmonary emboli. Enoxaparin was given for inpatient management, and she was discharged home a month later with apixaban 5mg twice daily. Follow-up coagulopathy screening yielded negative results, ruling out the presence of an underlying condition that would have contributed to her thrombotic complications.

## Discussion

This unique case demonstrates the potential for severe intestinal ischemia and necrosis necessitating bowel resection in the setting of post-COVID-19-induced coagulopathy. Severe COVID-19 infection may be linked to the development of GI ischemia, but symptom presentation varies greatly, from asymptomatic to deteriorating systemic status [4]. The onset of abdominal pain and worsening diarrhea two weeks after this patient’s initial COVID presentation and five days after the resolution of her respiratory symptoms suggest that the COVID-induced hypercoagulable state may persist long after the resolution of COVID symptoms. However, few cases of mesenteric ischemia have been reported in individuals recovered from COVID infection [5, 6]. A review of 89 patients found eight cases of mesenteric ischemia in patients on anticoagulation after a prior hospitalization for COVID-19 pneumonia [7]. Another review discovered no comorbidities in 13 of 41 cases of mesenteric ischemia in COVID-19 patients [8]. Emerging research documenting the relationship between mesenteric ischemia and COVID-19, combined with the absence of predisposing factors for thrombus formation in this patient, supports a causal relationship between this patient’s mesenteric ischemia and prior COVID-19 infection.

The incidence of mesenteric ischemia in critically ill patients with COVID-19 is relatively low, ranging between 3.8% and 4%, according to a review of the literature by Sunil Basukala [9]. However, the importance of early identification of mesenteric ischemia cannot be overstated, as it is one of the deadliest sequelae associated with COVID-19. The presence of a large ovarian mass in this patient distracted physicians and delayed the ultimate diagnosis of intestinal ischemia. It is important that life-threatening diagnoses such as mesenteric ischemia are ruled out early on since the overall mortality of patients with COVID-19 and GI ischemia ranges from 38%-54% [7,9]. Furthermore, mesenteric ischemia is difficult to diagnose since early lab values and imaging studies are often normal [4,10].

Activation of the immune system and inflammatory signaling cascades is hypothesized to be one of the main drivers of thrombogenesis in COVID-19 [4]. Some studies theorize that the etiology of COVID-19 thrombogenesis is due to direct activation of the coagulation cascade, given its similarity to SARS-CoV [11,12]. Regardless, numerous studies postulate that elevations in D-dimer and fibrin/fibrinogen degradation products have been associated with worse clinical outcomes and may be a useful marker in the identification of thromboembolic diseases, such as mesenteric ischemia [11,13,14]. This suggests that expanding the initial laboratory workup in patients with COVID-19 and abdominal pain may lead to better clinical outcomes. The recommended laboratory work-up includes a complete blood count (CBC), comprehensive metabolic panel (CMP), ABG, D-dimer, C-reactive protein (CRP), and lactic acid levels to evaluate for early signs of ischemia such as acidosis and elevated inflammatory markers. However, a normal lactic acid level or unremarkable imaging such as CT does not rule out the presence of mesenteric ischemia. Because of the high mortality and diagnostic challenges associated with mesenteric ischemia, it is imperative that physicians obtain a thorough history and physical exam and maintain a high index of suspicion for the disease in COVID-19 patients presenting with predominately GI complaints.

## Conclusions

This report highlights a rare case of severe thromboembolic complications following a prior COVID-19 diagnosis in a patient who recuperated at home. The individual remained asymptomatic for five days post-recovery before developing mesenteric ischemia and subsequent bowel necrosis. Moreover, her case was complicated by a lack of ischemic evidence from laboratory and imaging studies. The unforeseen nature of COVID-19 and its complications, such as mesenteric ischemia, present significant challenges to physicians due to the lack of ischemic evidence that may not appear in early laboratory values and imaging studies. A timely diagnosis of mesenteric ischemia is imperative given its high mortality rate. Furthermore, the COVID-induced hypercoagulable state may persist much longer than the initial symptom presentation, necessitating physicians to take a thorough history and physical exam and maintain a high index of suspicion for mesenteric ischemia.
